# Supplementary material for: Racism in public health authorities–A scoping review and situational analysis
Source: PLOS Glob Public Health. 2025 Nov 24;5(11):e0004350. doi: 10.1371/journal.pgph.0004350 (PMC12643306; doi:10.1371/journal.pgph.0004350)
Supplement: Attachment — Submitted filename: Response to Reviewers.docx [file pgph.0004350.s005.docx]

PGPH-D-25-00327
Racism in public health authorities – a scoping review and situational analysis
PLOS Global Public Health

Dear Dr. Razum,

Thank you for submitting your manuscript to PLOS Global Public Health. After careful consideration, we feel that it has merit but does not fully meet PLOS Global Public Health’s publication criteria as it currently stands. Therefore, we invite you to submit a revised version of the manuscript that addresses the points raised during the review process.

Both reviewers have indicated the substantial value of your review and provided focal comments to be addressed in the Introduction, Methods, and Discussion sections, which would be beneficial to the final manuscript. In addition to Reviewer 1's helpful requests for clarifications, Reviewer 2 makes several important constructive suggestions throughout the manuscript, all of which appear to be relatively straightforward to address. 

Please submit your revised manuscript by May 26 2025 11:59PM. If you will need more time than this to complete your revisions, please reply to this message or contact the journal office at globalpubhealth@plos.org. When you're ready to submit your revision, log on to [https://www.editorialmanager.com/pgph/](https://deref-web.de/mail/client/qUl-feqYvf0/dereferrer/?redirectUrl=https%3A%2F%2Fwww.editorialmanager.com%2Fpgph%2F) and select the 'Submissions Needing Revision' folder to locate your manuscript file.

Please include the following items when submitting your revised manuscript:

- A rebuttal letter that responds to each point raised by the editor and reviewer(s). You should upload this letter as a separate file labeled 'Response to Reviewers'.
- A marked-up copy of your manuscript that highlights changes made to the original version. You should upload this as a separate file labeled 'Revised Manuscript with Track Changes'.
- An unmarked version of your revised paper without tracked changes. You should upload this as a separate file labeled 'Manuscript'.

Guidelines for resubmitting your figure files are available below the reviewer comments at the end of this letter.

We look forward to receiving your revised manuscript.

Kind regards,

Peter A. Newman, Ph.D
Academic Editor
PLOS Global Public Health

Journal Requirements: The authors’ answers are marked in yellow

1. We have amended your Competing Interest statement to comply with journal style. We kindly ask that you double check the statement and let us know if anything is incorrect.

Thank you, the revised statement is correct.

2. Please provide separate figure files in .tif or .eps format.

Our only figure is a text-based diagram which we cannot provide in .tif or .eps format.

For more information about figure files please see our guidelines:

[https://journals.plos.org/globalpublichealth/s/figures](https://deref-web.de/mail/client/ujrd5Ri_71E/dereferrer/?redirectUrl=https%3A%2F%2Fjournals.plos.org%2Fglobalpublichealth%2Fs%2Ffigures)

[https://journals.plos.org/globalpublichealth/s/figures#loc-file-requirements](https://deref-web.de/mail/client/HG1ESzGCzR8/dereferrer/?redirectUrl=https%3A%2F%2Fjournals.plos.org%2Fglobalpublichealth%2Fs%2Ffigures%23loc-file-requirements)

3. Please review your reference list to ensure that it is complete and correct. If you have cited papers that have been retracted, please include the rationale for doing so in the manuscript text, or remove these references and replace them with relevant current references. Any changes to the reference list should be mentioned in the rebuttal letter that accompanies your revised manuscript. If you need to cite a retracted article, indicate the article’s retracted status in the References list and also include a citation and full reference for the retraction notice.

We have not cited any retracted papers. We have made sure that the reference list follows the PLoS format by using the respective EndNote output style. We have added one reference to the text and the list (now number [30]):
Fleming, P. J., Spolum, M. M., Lopez, W. D., & Galea, S. (2021). The Public Health Funding Paradox: How Funding the Problem and Solution Impedes Public Health Progress. *Public Health Reports®*, *136*(1), 10-13. https://doi.org/10.1177/0033354920969172

5. Review Comments to the Author

Dear Reviewers,

We would like to sincerely thank you for your thoughtful and constructive feedback. Your comments were invaluable in helping us clarify key aspects of our methodological approach, strengthen our reflection on researcher positionality, and deepen the discussion of our findings in relation to the existing literature.

We have carefully addressed each of your suggestions in the revised manuscript and expanded relevant sections to reflect your input. We hope the revisions contribute to a clearer and more robust presentation of our work.

Thank you again for your generous and insightful engagement with our manuscript.

| **Reviewers’ comments** | **Response of the authors** |
| --- | --- |
| **Reviewer 1** | |
| Thank you for this interesting and important scoping review. It is well-written, with excellent structure and a strong methodology. The findings have important implications for health authorities. | Thank you for your positive and encouraging feedback. |
| Was grey literature searched in this review, e.g. reports, blogs? If not, why not? A sentence justifying the exclusion of this form of knowledge from the scoping review (especially given how racialisation shapes whose knowledge counts and access to academic knowledge production) would be important to add to the methods. | We have now added a sentence to the methods section justifying the exclusion of grey literature, which exceeded the scope of our review. |
| In a paper on a topic like this, it is important for the authors to reflect on their positionality in the methods section, especially their racial and ethnic backgrounds and how this shaped the focus on this topic. | Thank you for highlighting this. We have now included a positionality statement in the methods section. |
| Reviewer 2 | |
| I appreciated the opportunity to review your manuscript. I have offered comments in the spirit of strengthening your paper. The scoping review addresses an important and timely topic of racism in public health agencies. It is overall well-written. | Thank you very much, we appreciate your thoughtful and constructive feedback. |
| Abstract:  - could benefit from a brief definition or at least mention of racism as a structural determinant of health (as you have done in the introduction to the paper).  - "have been neglected as relevant contexts" - Change to institutional contexts. The use of contexts on its own is vague. consider different wording.  - What scoping review methodology was used? Suggest that you name briefly in the abstract | Adjusted as suggested. |
| Introduction  - strong overview of racism as a structural determinant of health and PHAs.  - Can you elaborate a bit more on how you arrived at the gap involving PHAs (is this simply a gap in the peer reviewed literature that has received little to no attention? Is this because less is published on PHAs relative to health care organizations where funding for health (or illness) is disproportionately directed?). Given PHAs' mandate, they should conceivably be more concerned with equity, social justice and racism. | Unfortunately, the evidence on the underlying reasons is unclear, which might also relate to the publication bias you referred to in one of your following comments. We added that this gap can be the result of several factors, including disproportionate resource allocation to PHA and health equity. |
| Research questions are clear - did the authors predominantly engage with structural forms of racism (i.e. institutional/systemic) or also interpersonal and individual as well? (from the discussion section, it appears that you were quite inclusive) | We have clarified in line 164f to reflect inclusive engagement with various forms of racism. |
| Methods - overall this section is clearly detailed. I appreciated the application of both a narrative synthesis and situational analysis | Thank you, we appreciate your support. |
| Briefly, what approach to a scoping review did you use? needs to be more explicit. Here are two commonly used references: <https://www.tandfonline.com/doi/abs/10.1080/1364557032000119616> and <https://link.springer.com/article/10.1186/1748-5908-5-69> | We specified the approach here and in the abstract, based on Grant & Booth 2009, which was referenced in the publications suggested by reviewer 2. |
| search strategy - the search strategy is comprehensive. It's unclear what the publication dates were used to delimit the search (articles included were published between X date and Y date and why this timeframe was selected) | As described in lines 172/173, we did not limit our search to specific publication dates. |
| Given that some organizations may offer both preventive and curative services, how did you contend with such cases? Were such articles excluded? | Our eligibility criteria pertained to a) the setting of PHA and b) (anti-)racism. The type of public health services was not part of the eligibility criteria, but rather an analytical element. We excluded analyses of racism in healthcare facilities or racialized health inequalities without any explicit explanation of the role of PHA (see lines 170-171). |
| Results & discussion  in the opening line to this section, it would be helpful to briefly mention how many were originally considered. Of the XXX that were identified through our search strategy, we included XX after XXX screening, and then 55 publications after full text screening. | Added as suggested. |
| Are you able to summarize whether the focus of PHA featured in the articles included was at the national, sub-national (e.g. local or regional) or international level? | We have extracted this information in the data charting. However, we are hesitant to add this information to table 1 which is already extensive. We have described the information in the text for most of the analysis. |
| Conceptualization of racism - for the papers that did define racism, did any reference social theory (e.g. critical race theory) in their analyses? If yes, which theories or frameworks? If not, this may be worth mentioning as an analytic point in the discussion and future work | For those who gave a definition of racism, we gave direct quotes in Table 1 – most of these did not make explicit reference to any social theory when defining racism. We elaborated on these finding in lines 248-257. We highlighted the theories and frameworks for conceptualizing racism in PHAs in Table 2 (Major issues, debates). |
| I appreciated your analysis of how PHAs socially constructed high risk groups resulting in othering and also homogenizing population groups (e.g. Latinx). Another term that has received considerable attention is 'vulnerable'. It showed up in your results as well (the critique is that it puts the blame on the populations rather than drawing attention to the structures that make populations vulnerable - some have shifted the use of language away from vulnerable to populations made structurally vulnerable. Did this emerge in your analysis as well? | Excellent point, thank you for highlighting the need to use inclusive language. For our analysis and interpretation, we used the terminology of marginalized populations to emphasize the societal processes. We adjusted the terminology used in the articles included as suggested. |
| In presenting your results, you implicitly refer to different functions of PHAs (e.g. surveillance, programs, policies, guidelines, legal authority/oversight). What were the gaps in terms of PHA functions. For example, health promotion (which is part of the remit of PHA) wasn't mentioned but likely a limitation of what was in your included sample of articles | We added a sentence in the discussion to emphasize the lack of health promotion as a public health service. |
| Table 2 is excellent - here you do give some useful examples of PHAs operating at different levels (see comment above). I appreciate the challenges given the limited inclusion of potentiality statements. This may point to the likely omission of using theory/theoretical frameworks (see earlier comment) | See response above. |
| Strengths and Limitations are well outlined. Is it possible that there may be a publication bias (less written on PHAs relative to health care orgs), recognizing the challenges with the definition of public health services. | Thank you for this thought. We do not think the problem of defining PHA leads to a publication bias – such a bias would be introduced for example if only reports from PHA finding evidence of racializing would be published (as compared to findings of no such evidence). |
| Implications section focuses on practice - are there also any implications for future research that your findings should point to? | Yes, those implications also pertain to research, so we slightly expanded the implications section to reflect this. |
| Minor points  line 236 - roles should be plural  line 425 - the findings might reflect on the on the contested relationship - delete 'on the' which appears twice - | Corrected as suggested. |
